# Exosomes from adipose tissue-derived mesenchymal stem cells ameliorate histone-induced acute lung injury by activating the PI3K/Akt pathway in endothelial cells

**DOI:** 10.1186/s13287-020-02015-9

**Published:** 2020-11-27

**Authors:** Yukie Mizuta, Tomohiko Akahoshi, Jie Guo, Shuo Zhang, Sayoko Narahara, Takahito Kawano, Masaharu Murata, Kentaro Tokuda, Masatoshi Eto, Makoto Hashizume, Ken Yamaura

**Affiliations:** 1 Department of Disaster and Emergency Medicine, Graduate School of Medical Sciences, Kyushu University, 3-1-1 Maidashi, Higashi-ku, Fukuoka, 812-8582 Japan; 2 Department of Anesthesiology and Critical Care Medicine, Graduate School of Medical Sciences, Kyushu University, Fukuoka, Japan; 3 Emergency and Critical Care Center, Kyushu University Hospital, Fukuoka, Japan; 4 Center for Advanced Medical Innovation, Kyushu University, Fukuoka, Japan; 5 Intensive Care Unit, Kyushu University Hospital, Fukuoka, Japan

**Keywords:** Sepsis, Acute lung injury, Acute respiratory distress syndrome, Histones, Endothelial damage, Adipose-derived mesenchymal stem cells, Exosomes, PI3K/Akt signaling pathway, miR-126

## Abstract

**Background:**

Mesenchymal stem cells (MSCs), including adipose-derived mesenchymal stem cells (ADSCs), have been shown to attenuate organ damage in acute respiratory distress syndrome (ARDS) and sepsis; however, the underlying mechanisms are not fully understood. In this study, we aimed to explore the potential roles and molecular mechanisms of action of ADSCs in histone-induced endothelial damage.

**Methods:**

Male C57BL/6 N mice were intravenously injected with ADSCs, followed by histones or a vehicle. The mice in each group were assessed for survival, pulmonary vascular permeability, and histological changes. A co-culture model with primary human umbilical vein endothelial cells (HUVECs) exposed to histones was used to clarify the paracrine effect of ADSCs. Overexpression and inhibition of miR-126 ADSCs were also examined as causative factors for endothelial protection.

**Results:**

The administration of ADSCs markedly improved survival, inhibited histone-mediated lung hemorrhage and edema, and attenuated vascular hyper-permeability in mice. ADSCs were engrafted in the injured lung and attenuated histone-induced endothelial cell apoptosis. ADSCs showed endothelial protection (via a paracrine effect) and Akt phosphorylation in the histone-exposed HUVECs. Notably, increased Akt phosphorylation by ADSCs was mostly mediated by exosomes in histone-induced cytotoxicity and lung damage. Moreover, the expression of miR-126 was increased in exosomes from histone-exposed ADSCs. Remarkably, the inhibition of miR-126 in ADSCs failed to increase Akt phosphorylation in histone-exposed HUVECs.

**Conclusion:**

ADSC-derived exosomes may exert protective effects on endothelial cells via activation of the PI3K/Akt pathway.

## Background

Sepsis, which occurs as a result of severe infection, often leads to systemic dysregulated host response and remains a common cause of morbidity and mortality in intensive care units despite appropriate therapies [1]. Acute respiratory distress syndrome (ARDS) is a devastating complication of severe sepsis, and thus far, there are no direct therapies for it. Previous studies have shown that stem cells have multiple beneficial properties and are therapeutic agents for ARDS and sepsis [2–6]. Adipose-derived mesenchymal stem cells (ADSCs), a population of pluripotent mesenchymal stem cells (MSCs), are one of the most promising types of stem cells used as therapy [7, 8]. MSCs can attenuate organ damage owing to their capacity of homing to injured tissue [9] and secrete paracrine signals in ARDS and sepsis [5, 6, 10]. Furthermore, MSC-derived exosomes, microvesicles with a side of 30–120 nm, which contain various macromolecules, have been suggested to be one of the most important paracrine factors that play an essential role in this process [6, 11–13]. Compared to bone marrow-derived MSCs, ADSCs are more easily and abundantly obtained by minimally invasive methods [14]. Therefore, ADSCs and ADSC-exosomes have potential clinical applications. However, the precise mechanisms underlying ADSC therapy for ARDS and sepsis remain unclear. An elucidation of this mechanism would be beneficial for the development of stem cell-based therapy in the future.

Recent studies have reported that under conditions of sepsis, extracellular histones released from dead cells, including neutrophil extracellular traps (NETs) and damage-associated molecular patterns (DAMPs), induce endothelial damage in a concentration-dependent manner [15] and cause multiple organ failure (MOF) [16, 17]. Moreover, in ARDS, histones are believed to primarily contribute to the onset and propagation of acute lung injury [5, 18]. Although the mechanisms underlying histone-related cytotoxicity are not fully understood, apoptosis via the Akt pathway in endothelial cells is one of the outcomes [15, 19]. For this reason, therapies that inhibit the release of histone or histone-related signal transduction are supposed to significantly protect endothelial cells from damage, and thus could be a novel therapeutic approach for ARDS and sepsis [20, 21].

Histone administration is a newly established model for investigating the mechanisms of endothelial damage according to circulating extracellular histones in mice [21–23] and primary human umbilical vein endothelial cells (HUVECs) [15]. Although ADSCs have been reported to prevent lipopolysaccharide (LPS) [24] and cecal ligation and puncture (CLP)-induced ARDS and sepsis models [3, 6], their role in endothelial prevention in histone-induced models has not been elucidated. Therefore, we aimed to elucidate the effect of ADSCs and their exosomes on histone-induced endothelial damage in vivo and in vitro.

## Materials and methods

### Animals

Male C57BL/6N mice of 9–10 weeks of age, weighing 20–25 g and purchased from Charles River Laboratories Japan, Inc. (Yokohama, Japan), were used in this study. The mice were kept in controlled chambers (22 °C ± 2 °C and 12-h light/dark cycle) and provided with water and food ad libitum. The Ethics Committee for Animal Experiments at Kyushu University approved all animal experiments (project number A29-302-0). All the methods used were in accordance with the Guidelines for Animal Experiments of Kyushu University.

### Preparation and characteristics of ADSCs

Mouse ADSCs were collected from the inguinal fat pad. Adipose tissues were washed with phosphate-buffered saline (PBS) to remove residual blood. The tissues were cut into 1 × 1 mm pieces and digested in 1 mg/mL collagenase type II (Sigma-Aldrich, St. Louis, MO, USA) at 37 °C for 1 h. After centrifugation, the resulting cell pellet was resuspended in Dulbecco’s modified Eagle’s medium (DMEM) and filtered through a 70-μm nylon filter (Thermo Fisher Scientific, Waltham, MA, USA), washed four times, and then cultured in STK1 and STK2 medium (TWOCELLS COMPANY, Hiroshima, Japan) and antibiotic-antimycotic (Gibco, Thermo Fisher Scientific, Waltham, MA, USA) in a humidified atmosphere with 5% CO_2_ at 37 °C. Cells from the fifth passage were used. Cell immunophenotypes were analyzed using a flow cytometer EC800 (Sony, Tokyo, Japan). Briefly, cells were incubated with fluorescein-coupled antibodies against CD29, CD34, CD44, CD45, or CD90 at 4 °C for 1 h (BD Biosciences, San Jose, CA, USA). Isotype-identical antibodies served as controls (Additional file 1: Figure S1a).

### Exosome isolation, analysis, and labeling check

Exosomes were isolated using the ExoQuick-TC exosome precipitation kit (System Biosciences, Mountain View, CA), according to the manufacturer’s specifications. ADSC culture medium was centrifuged at 3000×*g* for 15 min to remove dead cells and cellular debris. The supernatant (10 mL) was then mixed thoroughly with 2 mL of ExoQuick-TC reagent and stored at 4 °C overnight. The supernatant mixed with ExoQuick-TC solution was centrifuged at 1500×*g* for 30 min to obtain a pellet containing exosomes, which were then resuspended in 100 μL of PBS. The concentration of exosomes was measured using the bicinchoninic acid assay (BCA) protein assay kit. The protein levels of CD63, CD9, CD81 (representative markers of exosomes), and β-actin (negative control) were identified using western blotting (Additional file 1: Figure S1b). The size distribution (hydrodynamic diameter) of exosomes was determined by a dynamic light scattering technique using a Zetasizer Nano ZS analysis system (Malvern Instruments Ltd., Worcestershire, UK) (Additional file 1: Figure S1c).

### Exosome inhibition in ADSCs

For further validation of ADSC-exosomes, ADSCs were cultured with 20 μM GW4869 (inhibitor of neutral sphingomyelinase that prevents exosome release from ADSCs) (Sigma-Aldrich) or an equivalent volume of DMSO for 12 h; ADSCs were then used in in vivo and in vitro experiments. Equal amounts of protein were extracted from ADSC-exosomes and GW4869-treated ADSC-exosomes for western blotting to detect the expression of CD81 marker.

### Animal study design

For the histone infusion model, mice were intravenously injected with a total volume of 100 μL of calf thymus histones (Sigma-Aldrich) at 75 mg/kg body weight as previously described [21, 25, 26]. The control group was injected intravenously with 100 μL of saline solution. To assess the effects of ADSC treatment, ADSCs, or GW4869-treated ADSCs (3 × 10^5^ cells/mice), a total volume of 100 μL or 100 μL of PBS was intravenously injected 30 min prior to histone injection. Additionally, to assess the effects on the PI3K/Akt signaling pathway, mice were intraperitoneally injected 1 h prior to ADSC injection with LY294002 (Akt inhibitor) (Selleck Chemicals, Houston, TX, USA) at 40 mg/kg per 200 μL or 200 μL of PBS. Mice were monitored for survival for up to 6 h after histone injection. Mice that presented signs of severe distress, such as dyspnea and cyanosis, were euthanized by cervical dislocation, and these cases were recorded as histone-induced mortality. Mice were sacrificed 6 h after histone or saline administration, and their lung samples were harvested for analysis.

### In vivo micro-CT imaging

Tissue density of mouse lung imaging was performed using an in vivo micro-CT system (R_mCT2, RIGAKU, Tokyo, Japan). The image was acquired at 90 kV, 160 μA, and 20 × 20 mm field of view and a scan time duration of 2 min, with respiratory gating. The imaging procedures were carried out under isoflurane (3% for induction and 1.5% for maintenance).

### Histological examination

After fixing in 10% formalin, lung tissue was paraffin-embedded and cut into 6-μm sections. For histological assessment, the sections were stained with hematoxylin and eosin (H&E). The sections were visualized using Axio Scan Z1 (Carl Zeiss AG. Ltd., Thornwood, NY, USA) and Zen software (Carl Zeiss AG. Ltd.).

### Vascular permeability assay

Pulmonary microvascular permeability was measured using Evans Blue dye, as previously described [27]. Briefly, 200 μL of 0.5% Evans Blue dye (Nacalai Tesque, Inc. Kyoto, Japan) was injected intravenously 10 min prior to ADSC or PBS administration. Mice were injected with LY294002 and histones and euthanized at previously described time points. The lungs of sacrificed mice, which were perfused with 5 mL of PBS through their right ventricle and injected with histones after 1 h, was harvested, dried at 60 °C for 24 h, and then weighed. Evans Blue in the dried lungs was extracted by immersion in 3 mL of 100% formamide at 37 °C for 48 h. Evans Blue concentration in the supernatant was then determined by absorbance at 620 nm using a microplate reader (EnSpire 2300 Multilabel; PerkinElmer Inc., Waltham, MA, USA) and expressed as micrograms per gram of dry tissue weight.

### Immunofluorescence analysis of frozen tissue samples

We performed double immunofluorescence staining for CD31 and cleaved caspase-3 in lung sections. Briefly, samples were fixed in an optimal cutting temperature (OCT) compound and frozen in liquid nitrogen. Sections were cut into 10-μm sections, blocked with 10% goat serum (Abcam), and incubated with primary antibodies [CD31 rat monoclonal 1:200 (Abcam, Cambridge, UK) and cleaved caspase-3 rabbit polyclonal 1:200 (Cell Signaling Technology, Inc. Danvers, MA, USA)] in 1% bovine serum albumin (BSA) overnight at 4 °C, followed by secondary antibodies [Alexa Fluor 555 goat anti-rat and Alexa Fluor 488 goat anti-rabbit (Abcam) and Hoechst 33342 solution (Dojindo Laboratories, Kumamoto, Japan)] for nuclear staining for 30 min at ambient temperature. Sections were visualized under an FV-1000D confocal laser scanning microscope (Olympus, Tokyo, Japan).

To monitor the in vivo distribution of ADSCs, ADSCs were labeled with CellVue Claret Far Red Fluorescent Cell Linker Kits (Sigma-Aldrich) according to the manufacturer’s instructions. Labeled ADSCs were seeded in a six-well μ-slide (Ibidi, Munich, Germany) and visualized. Then, 4 × 10^6^ labeled ADSCs per mouse were intravenously injected. Lung sections were observed as described previously.

### HUVEC culture and experimental design

HUVEC cultures were obtained from Cell Applications, Inc. (San Diego, CA, USA). HUVECs were cultured in endothelial cell growth medium kit (Cell Applications) and collagen-coated flasks. HUVECs from passages 6 to 8 were used in this study. When they reached 80% confluence, the media were changed and cells exposed for 4 h to different calf thymus histone concentrations (Sigma-Aldrich): 25, 50, 75, or 100 μg/ml prepared in PBS. Non-contact co-culture of HUVECs and ADSCs was performed in cell culture inserts (24 wells, 0.4 μm pore size, Falcon, Franklin Lakes, NJ, USA). HUVECs (5 × 10^4^ cells/well) and ADSCs (1 × 10^4^ cells/well) were seeded into lower and upper chambers, respectively, in a 1:1 mixture of endothelial cell growth medium and STK2 medium. The ADSCs-GW4869 group was cultured with 20 μM GW4869 or an equivalent volume of DMSO in the ADSC-DMSO group for 12 h. Then, the lower and upper chambers were connected, and non-ADSC groups used the empty upper chamber. Cells were exposed to 100 μg/ml of histones for 4 h. Samples were analyzed using viability and protein assays. To evaluate cell viability, a luminescent ATP assay kit, Cell Titer-Glo luminescent cell viability assay (Promega, Madison, WI, USA), was employed. Luminescence was detected using a microplate reader (EnSpire 2300 Multilabel, PerkinElmer Inc.). Cell viability was calculated as a percentage normalized to that of the control group.

### Protein expression analysis

Tissue and HUVECs were homogenized in ice-cold Pierce RIPA Buffer (Thermo Fisher Scientific) containing protease and phosphatase inhibitor cocktail cOmplete EDTA-free (Sigma-Aldrich), phenylmethylsulfonyl fluoride (PMSF) (Sigma-Aldrich), and sodium orthovanadate (Wako, Tokyo, Japan). Protein concentration was determined using a Protein Quantification Kit-Rapid (Dojindo Laboratories). Equal amounts of proteins were separated via SDS-PAGE (Wako) and transferred to a membrane using Trans-Blot Turbo Transfer System (Bio-Rad, Hercules, CA, USA). The membranes were blocked with 5% skimmed milk and immunoblotted with primary antibodies that recognize one of the following targets at 4 °C overnight: CD63 (Abcam), CD9 (Abcam), CD81 (Cell Signaling Technology, Inc. Danvers, MA, USA), β-actin (Cell Signaling Technology), phospho-Akt (Ser473)(Cell Signaling Technology), Akt (Cell Signaling Technology), cleaved caspase-3 (Cell Signaling Technology), and caspase-3 (Cell Signaling Technology). Signals were detected with horseradish peroxidase-conjugated goat anti-rabbit IgG (Vector Laboratories, Burlingame, CA, USA) and visualized using chemiluminescence with the Clarity Western Enhanced chemiluminescence (ECL) substrate (Bio-Rad). ECL signals were digitized using ImageJ software version 1.50i (National Institutes of Health, Bethesda, MD, USA).

### miR-126 mimic and inhibitor transfection

For overexpression and inhibition of miR-126, miR-126-mimic (hsa-miR126-3p miRVana miRNA mimic, Cat# 4464066, Assay ID MC12841) and miR-126-inhibitor (hsa-miR126-3p miRVana miRNA inhibitor, Cat# 4464084, Assay ID MH12841) (Thermo Fisher Scientific) were diluted to a final concentration of 10 nM with transfection compounds. ADSCs were transfected using Lipofectamine RNAiMAX Transfection Reagent (Invitrogen Life Technologies, Waltham, MA, USA), following the manufacturer’s instructions. At 48 h after transfection, the resulting cells were used for experiments.

### RT-PCR analysis

Total RNA, including miRNA from ADSCs and exosomes, was isolated using the miRNeasy Mini Kit (Qiagen, Valencia, CA, USA) and the SeraMir Exosome RNA Purification Kit (System Biosciences) according to the manufacturer’s protocol. Complementary DNAs (cDNAs) were obtained using the Taqman MicroRNA Reverse Transcription Kit (Invitrogen). RT-PCR was conducted using the Taqman MicroRNA Assay (Invitrogen) and a real-time fluorescent quantitative PCR 7500 system (Applied Biosystems, Foster City CA, USA). The expression of hsa-miR-126 (Cat# 4427975, Assay ID 002228) was normalized to that of U6 snRNA (Cat# 4427975, Assay ID 001973) as an internal control for miRNAs. Fold changes were calculated using the 2−∆∆Ct method. miRNA from histone-exposed ADSCs and exosomes derived from histone-exposed ADSCs were compared to miRNA from the respective control conditions. Histone-exposed ADSCs were exposed to 100 μg/ml of histones for 2 h.

### Statistics

Data are presented as mean ± standard error of the mean (SEM). Differences among the groups were evaluated using the one-way analysis of variance (ANOVA) followed by Tukey’s multiple comparisons test, and two-sample Student’s *t* test using Prism 7.0a (GraphPad Software, La Jolla, CA). Kaplan-Meier survival analysis was performed using the log-rank test. *p* values lower than 0.05 were considered statistically significant.

## Results

### ADSCs improved survival rate and inhibited histone-mediated lung injury

**Fig. 1:**
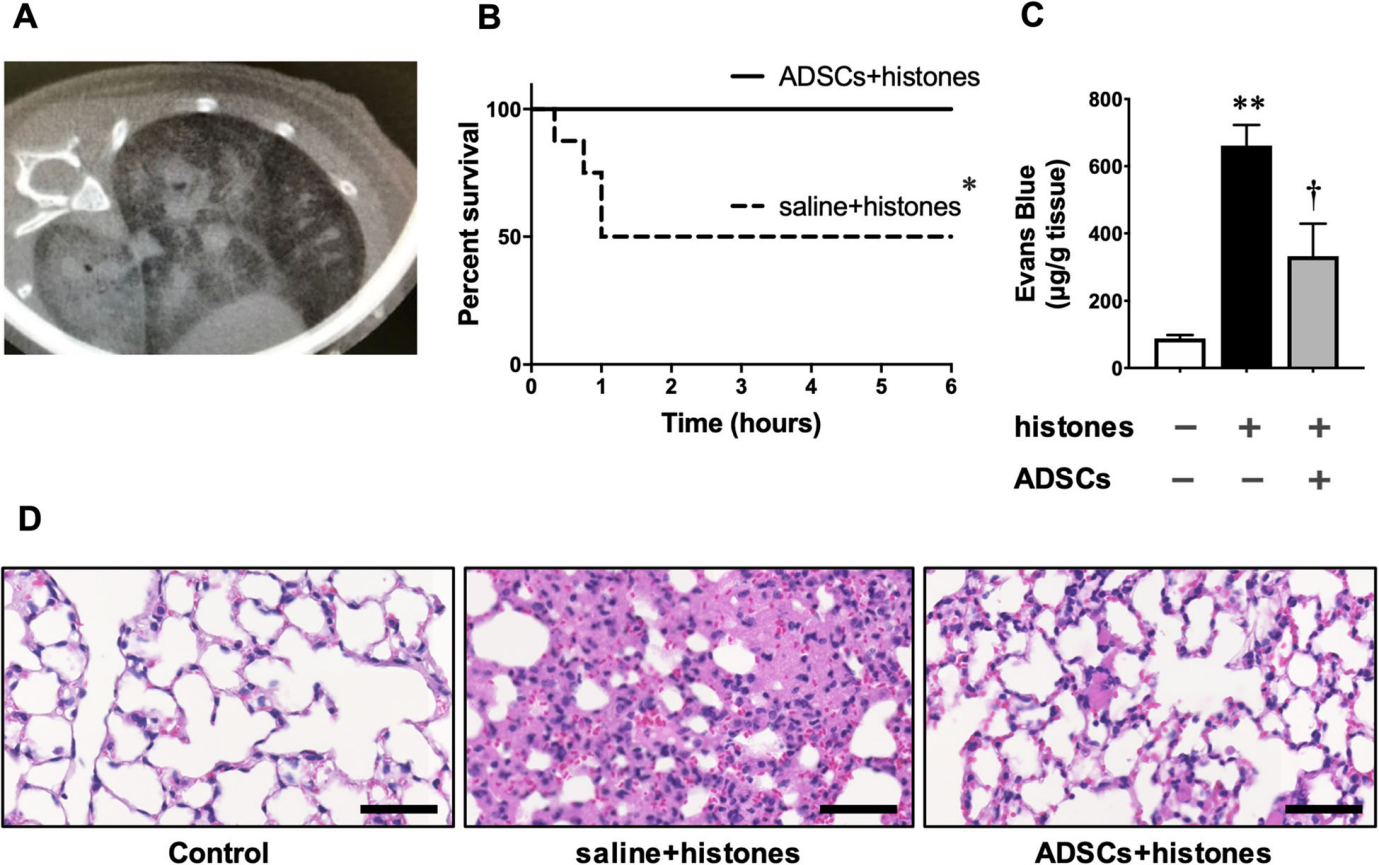
Effects of histones with or without ADSC administration in mice. **a** Representative pictures of the lung CT scan 1 h after injection of 75 mg/kg histones. **b** Kaplan-Meier survival curves showing survival in the saline+histones group and the ADSCs+histones group. *N* = 8 in each group. ^*^p = 0.0256 vs. saline+histones group. **c** In vivo pulmonary vascular permeability assay measured by Evans Blue dye. *N* = 5 in each group. ^**^p < 0.01 vs. control group, ^†^*p* < 0.05 vs. saline+histones group. **d** Representative photomicrographs of lung sections with H&E staining 6 h after injections of ADSCs and histones. Scale Bar, 50 μm

Administration of histones caused severe lung infiltrative shadows, especially around the bronchi, indicating lung hemorrhage and edema on computed tomography (CT) imaging (Fig. 1a). Four of the 8 mice (50%) died within 1 h after injection of histones. In contrast, all the mice injected with ADSCs and histones after 6 h remained alive (Fig. 1b, *p* = 0.0256 by log-rank test).

To evaluate vascular hyper-permeability, we performed an Evans Blue assay. We injected Evans Blue followed by the injection of histones with or without ADSCs. The vascular leak was significantly attenuated in the ADSCs+histones group compared to that in the saline+histones group (Fig. 1c). In the H&E staining, the result of which was consistent with that of the vascular permeability assay, the lungs of histone-infused mice showed congestion and edema and were concomitant with neutrophil infiltration in the alveolar capillaries, which was attenuated in the ADSCs+histones group (Fig. 1d).

### ADSCs attenuated histone-induced endothelial cell apoptosis in the lung

**Fig. 2:**
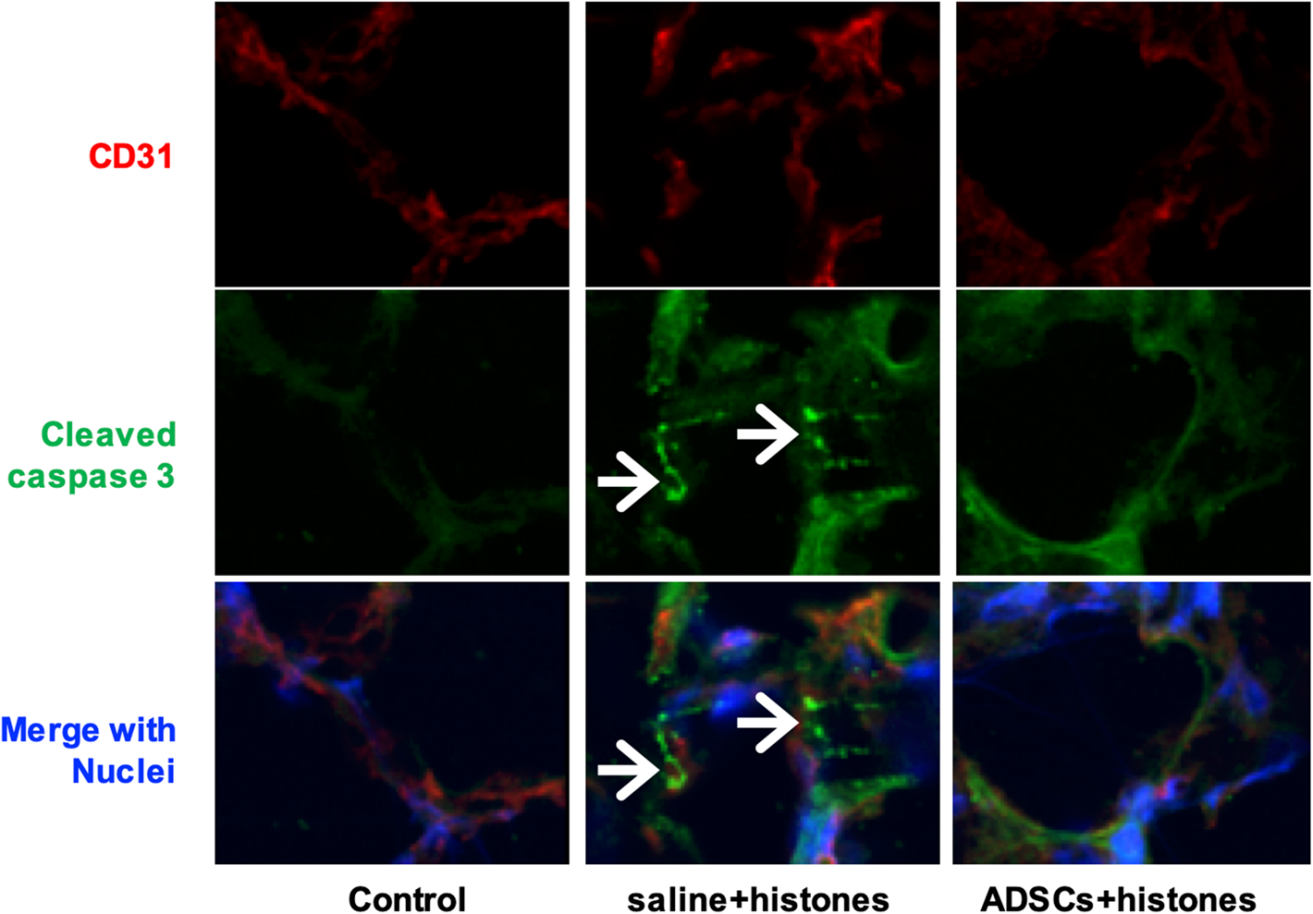
Immunofluorescent images in lung tissues. Cleaved caspase-3 (green, arrows) and endothelial cells are stained with anti-CD31 antibody (red). Overlap images indicate that the cleaved form of caspase-3 is present in endothelial cells. Nuclei were counterstained with Hoechst 33258 dye (blue)

To examine the effects of histones and ADSCs on the endothelial cells of the lungs, we performed multiple immunohistochemical analyses to detect cleaved caspase-3, an apoptotic marker, and CD31, an endothelial cell marker. Histones increased the signal for death of pulmonary endothelial cells, while ADSCs attenuated these signals (Fig. 2).

### Infused ADSCs engrafted in injured lung

**Fig. 3:**
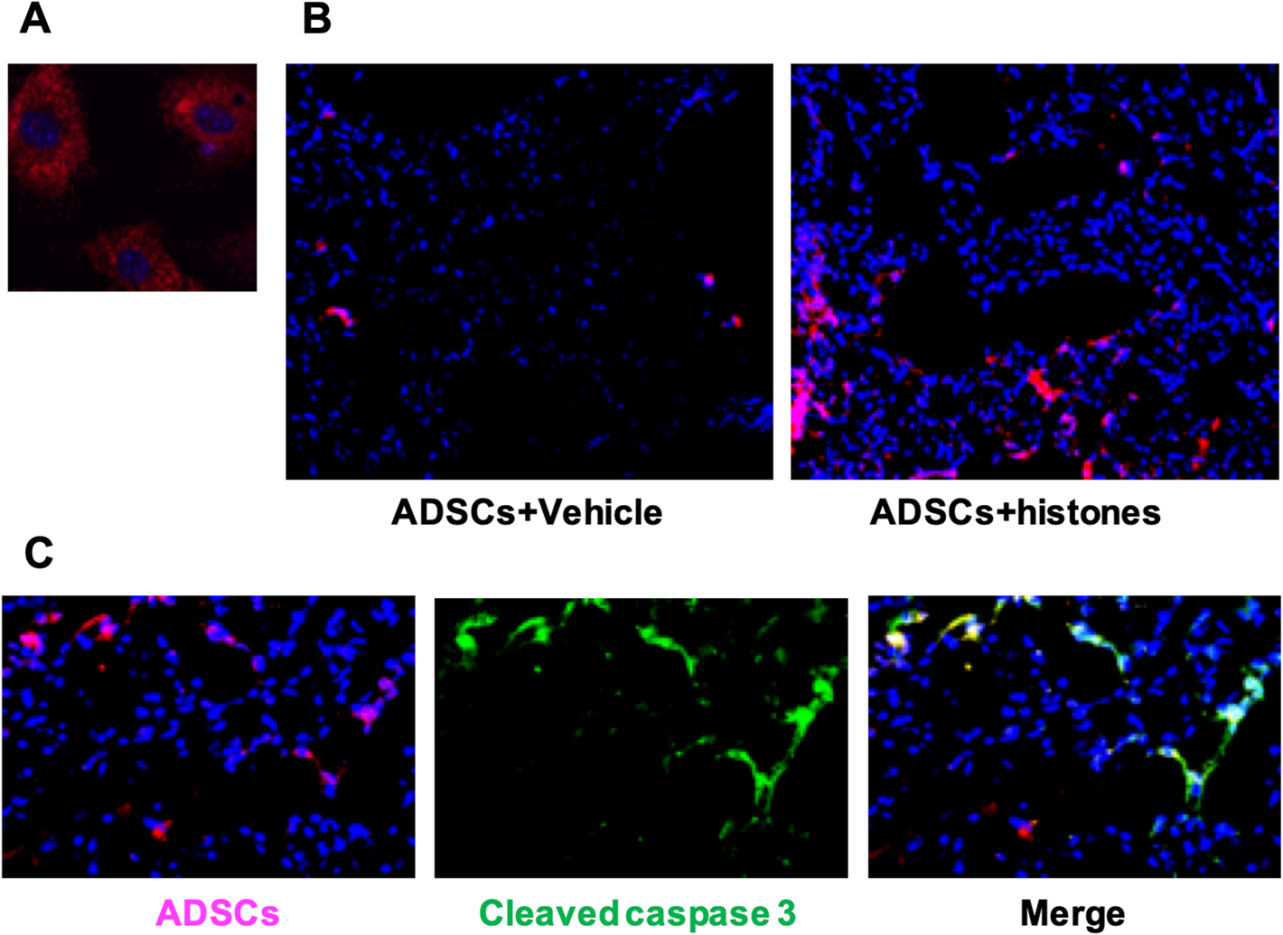
Fluorescent tracing of infused ADSCs. **a** Detection of red-fluorescent-labeled ADSCs using CellVue Claret Far Red Fluorescent Cell Linker Kits by fluorescence microscopy. Nuclei were counterstained with Hoechst 33258 dye (blue). **b** Cell Linker-labeled ADSCs were detected in the lung with or without histones. **c** Overlap images indicate that the cleaved form of caspase-3 (green) is present with Cell Linker-labeled ADSCs

To monitor the in vivo distribution of injected ADSCs, ADSCs labeled with CellVue Claret Far Red Fluorescent Cell Linker Kits were observed under a fluorescence microscope (Fig. 3a). Cell linker particles (red color) were present in the cytoplasm of ADSCs. Higher numbers of cell linker-labeled ADSCs were observed in the histone-injected lung than in the non-injected lung, 6 h after injection (Fig. 3b). We also found that engrafted ADSCs co-localized with the endothelial cells expressing the cleaved form of caspase-3, an apoptotic marker, in the histone-injected lung (Fig. 3c). Our results suggest that intravenously infused ADSCs have more potential to be engrafted in the injured lung than in the normal lung. These results are consistent with a previous report that ADSCs are capable of homing to injured tissues.

### ADSCs exerted an indirect endothelial protective effect via the paracrine effect of exosomes against histone-mediated cytotoxicity in endothelial cells

**Fig. 4:**
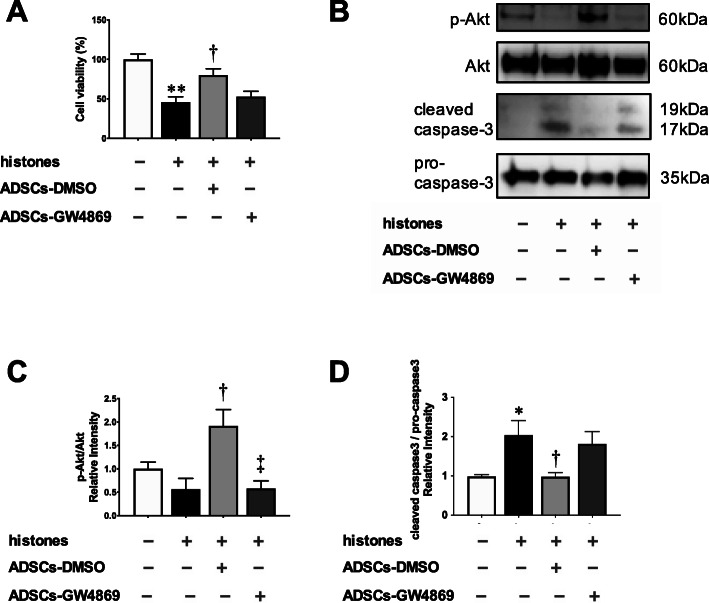
Paracrine effect of ADSCs against histone-mediated cytotoxicity with HUVECs. **a** The relative cell viability of HUVECs after co-culture with or without ADSCs-DMSO or ADSCs-GW4869 exposed to histones (100 μg/mL) for 4 h. *N* = 6 in each group. ^**^p < 0.01 vs. control, ^†^*p* < 0.05 vs. histones. **b**–**d** Representative immunoblots and densitometric analysis of phosphorylated Akt and cleaved caspase-3 in HUVECs 4 h after histone challenge with or without ADSCs-DMSO and ADSCs-GW4869. *N* = 4 in each group. ^*^p < 0.05 vs. control, ^†^*p* < 0.05 vs. histones, ^‡^*p* < 0.05 vs. histones+ADSCs-DMSO

The mechanism underlying the therapeutic effect of ADSCs is reported to include the direct differentiation of ADSCs into tissues and the indirect effect of secreted factors from ADSCs, called the paracrine effect. Since ADSCs presented an endothelial protective effect in 1 h, the protective effect of ADSCs was thought to be their paracrine effect. Therefore, we investigated the paracrine effect of ADSCs against histone-mediated cytotoxicity in an endothelial cell model. Histones reduced HUVECs viability in a dose-dependent manner (Additional file 1: Figure S1d). To investigate the paracrine effect, we cultured ADSCs in a non-contacting co-culture (Additional file 1: Figure S1e) with HUVECs exposed to histones. ADSCs significantly improved the viability of histone-exposed HUVECs in the non-contacting co-culture (Fig. 4a). This result reveals the paracrine effect of ADSCs against histone-exposed HUVECs.

Recently, studies have shown that exosomes secreted by ADSCs contribute to their paracrine effects in many diseases [28, 29]. To investigate the contribution of exosomes, we isolated putative exosome fractions from conditioned media of ADSCs and analyzed them. We also treated ADSCs with GW4869, an inhibitor of neutral sphingomyelinase that prevents exosome release [30, 31]. Exposure of ADSCs to 20 μM of GW 4869 for 12 h blocked exosome production (Additional file 1: Figure S1f). We cultured histone-exposed HUVECs for 4 h with ADSCs-DMSO or ADSCs-GW4869. Suppression of the release of exosomes abrogated the improved viability of ADSCs (Fig. 4a). In addition, ADSCs increased the phosphorylation of Akt and suppressed the cleavage of caspase-3, in the context of histone-mediated cytotoxicity toward HUVECs; however, suppression of the release of exosomes abrogated these effects (Fig. 4b–d). These results were consistent with those obtained in the context of the co-culture of exosomes with histone-exposed HUVECs; cell viability improved and Akt was phosphorylated (Additional file 1: Figure S1g-i). These data suggest that ADSC-derived exosomes may contribute to the paracrine effects on cell viability, accompanied by increased levels of phosphorylated Akt.

### ADSCs increased Akt phosphorylation and inhibition of PI3K/Akt pathway activation attenuated the protective effect of ADSCs on histone-mediated lung injury in mice

**Fig. 5:**
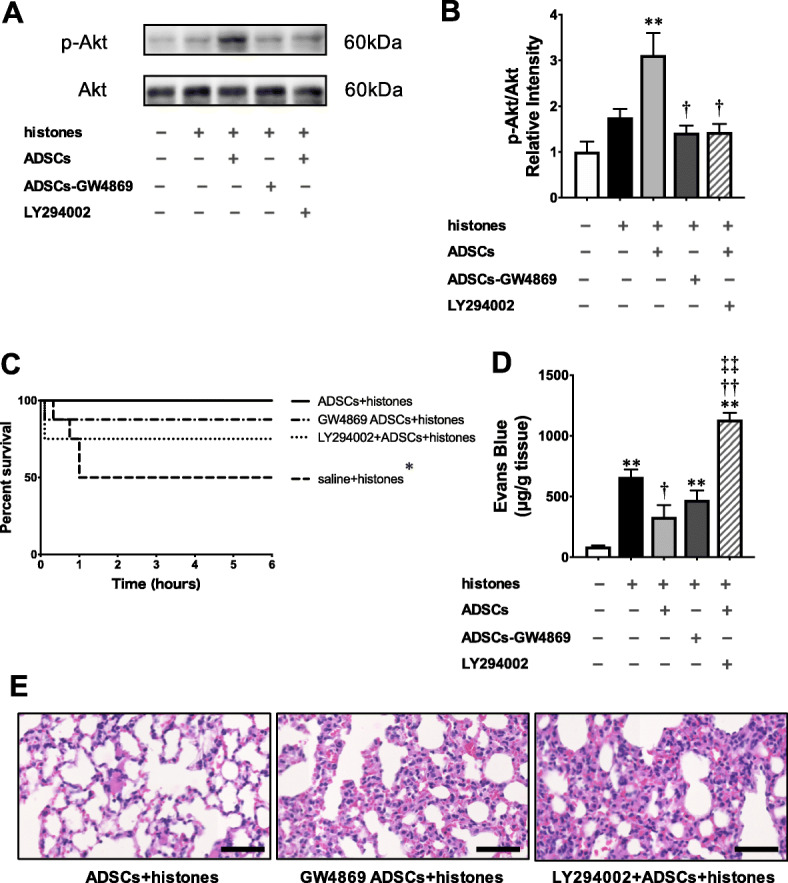
Inhibition of LY294002 and GW4869 on the protective effect of ADSCs in mice. **a**, **b** Representative immunoblots and densitometric analysis of phosphorylated Akt in lung tissue 6 h after histone challenge with or without ADSCs, ADSCs-GW4869, and LY294002. *N* = 5 in each group. ^**^p < 0.01 vs. control group, ^†^*p* < 0.05 vs. ADSCs+histones group. **c** Kaplan-Meier survival curves showing survival in the saline+histones group, the ADSCs+histones group, the GW4869 ADSCs+histones group, and the LY294002 + ADSCs+histones group. *N* = 8 in each group. **d** In vivo pulmonary vascular permeability assay measured by Evans Blue dye. *N* = 5 in each group. ^**^p < 0.01 vs. control group, ^†^*p* < 0.05 vs. saline+histones group, ^††^*p* < 0.01 vs. saline+histones group, ^‡‡^*p* < 0.01 vs. ADSCs+histones group. **e** Representative photomicrographs of lung sections with H&E staining 6 h after injections of ADSCs or ADSCs-GW4869 and histones with or without LY294002. Scale Bar, 50 μm

Further in vivo studies were performed to determine whether ADSCs and ADSC-exosomes could increase Akt phosphorylation. Lung tissues were harvested 6 h after injection of histones and processed for western blotting. Akt phosphorylation increased in the ADSCs+histones group compared to that in the control group; however, the exosome production-inhibited ADSCs (GW4869 ADSCs) group prevented it (Fig. 5a,b). Additionally, administration of LY294002 (an Akt inhibitor), before ADSCs inhibited ADSC-induced phosphorylation of Akt (Fig. 5a, b). The survival rate in the GW4869 ADSCs and LY294002 groups was slightly lower than that in the ADSCs+histones group (Fig. 5c). In the Evans Blue vascular permeability assay, GW4869 ADSCs, partially and LY294002 completely abolished the effects of ADSCs and significantly increased the vascular leak induced by histones (Fig. 5d). In H&E staining, the GW4869 ADSCs and LY294002 groups showed abolished attenuation of congestion and edema by ADSCs (Fig. 5e).

### miR-126 was significantly increased in histone-treated ADSCs and exosomes derived from histone-treated ADSCs

**Fig. 6:**
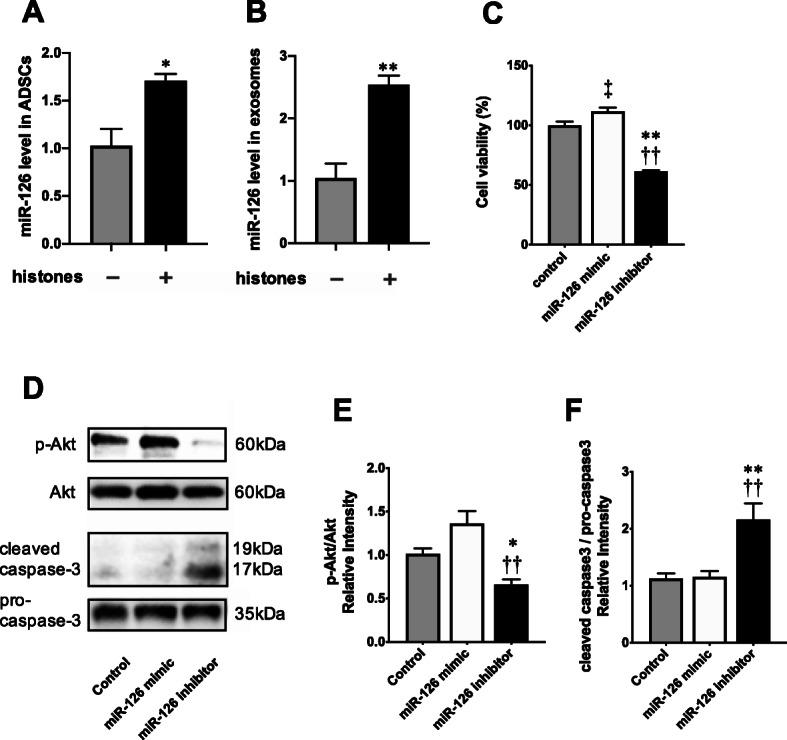
Inhibition of miR-126 on the protective effect of ADSCs against histone-mediated cytotoxicity with HUVECs. **a**, **b** The expression of miR-126 in ADSCs and exosomes before or after 2 h of exposure to histones was evaluated using RT-PCR. *N* = 3 in each group. ^*^p < 0.05, ^**^p < 0.01 vs. control group. **c** The relative cell viability of histone-exposed HUVECs (100 μg/mL, 4 h) after co-culture with ADSCs, miR-126 mimics ADSCs or miR-126 inhibitor ADSCs. *N* = 3 in each group. ^**^p < 0.01 vs. control, ^††^*p* < 0.01 vs. miR-126 mimic, ^‡^*p* < 0.05 vs. control. **d**–**f** Immunoblot results of phosphorylated Akt and cleaved capsase-3 in histone-exposed HUVECs (100 μg/mL, 4 h) co-cultured with ADSCs, miR-126 mimic ADSCs or miR-126 inhibitor ADSCs. *N* = 4~6 in each group. ^*^p < 0.05, ^**^p < 0.01 vs. control, ^††^*p* < 0.01 vs. miR-126 mimic

Next, we investigated the mechanism by which exosomes derived from ADSCs increased Akt phosphorylation. Recent studies have shown that miRNAs in exosomes play a role as key transporters in cell-cell micro-communication [29], and miR-126 has been recently reported as an essential messenger for endothelial angiogenesis and anti-apoptotic conditions [29, 32, 33]. To confirm the role of miR-126 in ADSCs and exosomes, the miR-126 expression levels in histone-exposed ADSCs and exosomes derived from histone-exposed ADSCs were compared to those in controls. RT-PCR analysis showed that miR-126 expression in histone-exposed ADSCs and exosomes was increased compared to that in the respective controls (Fig. 6a, b).

### miR-126-inhibiting ADSCs attenuated the viability and Akt phosphorylation of histone-exposed HUVECs

To further examine the effect of miR-126 on the PI3K/Akt pathway, miR-126 mimic and inhibitor were used in the context of ADSC culture to upregulate and downregulate miR-126 expression, respectively. The viability of histone-exposed HUVECs was enhanced and attenuated by miR-126-overexpressing ADSCs and miR-126-inhibited ADSCs, respectively (Fig. 6c). Additionally, the inhibition of miR-126 in ADSCs suppressed Akt phosphorylation and increased cleaved caspase-3 in histone-exposed HUVECs (Fig. 6d–f). These results suggest that miR-126 from ADSCs contributes to the activation of PI3K/Akt signaling and suppresses endothelial apoptosis.

## Discussion

To the best of our knowledge, this is the first study to demonstrate that ADSCs can rescue lethal histone-induced pulmonary injuries via endothelial protection. In the present study, the underlying mechanisms were examined using a histone-induced damage model in HUVECs. First, we found that the administration of ADSCs markedly improved survival and inhibited histone-mediated lung hemorrhage and edema in an in vivo study. Importantly, intravenously injected ADSCs were mostly engrafted in the injured lung and attenuated histone-induced endothelial cell apoptosis. In addition, ADSCs exerted an indirect endothelial protective effect via the paracrine effect of exosomes against histone-exposed HUVECs involving Akt phosphorylation. Akt phosphorylation was upregulated by ADSCs in histone-mediated injured lungs in mice. However, the protective effect via Akt phosphorylation failed in the exosome-inhibited ADSCs (GW4869-treated ADSCs) and Akt inhibitor (LY294002) groups (Figs. 4 and 5). Moreover, we examined the relationship between miR-126 and endothelial protection. The expression of miR-126 in histone-exposed ADSCs and derived exosomes was increased compared to that in controls. As expected, Akt phosphorylation decreased in the miR-126-inhibited ADSC-treated group compared to that in the control group. The viability of histone-exposed HUVECs was enhanced and attenuated by miR-126-overexpressing ADSCs and miR-126-inhibiting ADSCs, respectively. These results suggest that ADSC-derived exosomes containing miR-126 could contribute to paracrine effects against histone-induced endothelial damage accompanied by activation of the PI3K/Akt pathway.

In sepsis and sepsis-related ARDS, extracellular histones are released not only from dead cells but also from NETs and DAMPs, eventually leading to MOF and death in animal models [16, 17, 23]. In humans, increased levels of circulating histones have also been recognized in patients with sepsis and ARDS depending on their severity [34, 35]. Recently, some studies have shown that extracellular histones are cytotoxic to the endothelium in vitro [15] and cause lethal intra-alveolar hemorrhage in mice [21–23, 36]. Therefore, extracellular histones are increasingly becoming a concern and examined as a therapeutic target for sepsis and ARDS. Besides, there is no effective anti-histone therapy in clinical settings [20, 21]. Although the mechanisms underlying histone-related endothelial cytotoxicity are not fully understood, it has been reported that histones can cause direct endothelial cytotoxicity by integrating into the phospholipid bilayer of cell membranes, altering their permeability, thus resulting in an influx of calcium ions and cell death [17, 37, 38]. Histones also interact with Toll-like receptors (TLRs) and pro-inflammatory cytokines/chemokines, released through MyD88, NFκB, and NLRP3 inflammasome-dependent pathways [17, 21]. In previous studies, extracellular histone-treated HUVECs decreased eNOS expression [39], suppressed the Akt signaling pathway [15], and eventually induced apoptosis in a dose-dependent manner. In many animal models of sepsis, endothelial cell damage is associated with apoptosis [19, 40, 41]. Additionally, improvement of the PI3K/Akt signaling pathway attenuated apoptosis and improved survival in animal models of sepsis [41–43]. ADSCs and exosomes have the potential to trigger the PI3K/Akt signaling pathway in wound healing [44] and myocardial ischemia/reperfusion injury [45]. Therefore, we speculate that the PI3K/Akt signaling pathway is most closely related to histone-induced endothelial cytotoxicity and investigated the association between the PI3K/Akt pathway, histone-induced endothelial damage, and ADSC therapy.

The mechanism underlying the therapeutic effect of ADSCs is reported to include the direct differentiation of ADSCs into tissues and endothelial cells [46], the indirect effect of ADSCs homing to injured tissues and secreted paracrine signals through cell-cell contact [5, 9, 10]. Earlier studies have shown that a number of proteins, including vascular endothelial growth factor (VEGF), hepatocyte growth factor (HGF), and basic fibroblast growth factor (FGF2), play a role in paracrine effects of ADSCs [10, 47]. However, recent studies have shown that exosomes secreted by ADSCs also play a key role in paracrine mechanisms and have attracted attention in basic research and clinical applications [44]. In the present study, ADSCs showed an endothelial protective effect and improved murine survival in only 1 h. Therefore, we conjectured that this effect of ADSCs is due to the paracrine effect of ADSCs. We investigated the paracrine effect of exosomes in in vivo and in vitro models using GW4869 (inhibitor of exosome release). GW4869 decreased the endothelial protective effects of ADSCs. However, GW4869-treated ADSCs did not completely impact the survival of histone-induced mice. Thus, we speculate that the paracrine effect of exosomes is one part of the protective effects of ADSCs, but is a major part of the mouse model of histone-induced endothelial damage. Exosomes have been reported to contain messages in the form of mRNA, DNA, miRNAs, and proteins, including cytokines and growth factors that are transferable to target cells. Of these, miRNAs in exosomes have been reported as the most important micro-communication agents between cells [29]. Recent studies have shown that miR-126 is expressed in vascular endothelial cells and vascular smooth muscle cells and plays an important role in the process of angiogenesis through the regulation of cell proliferation, differentiation, and apoptosis [32, 33]. Moreover, miR-126 is essential for endothelial differentiation in ADSCs [48]. When miR-126 is downregulated, the PI3K/Akt signaling pathway is inhibited, affecting angiogenic factor signals and disrupting angiogenesis in HUVECs [49]. In this study, we investigated the mechanisms behind the exosome-mediated activation of the PI3K/Akt pathway and examined the influence of miR-126. The expression of miR-126 in histone-exposed ADSCs and in the respective exosomes was increased compared to that in controls. Additionally, miR-126-inhibiting ADSCs suppressed Akt phosphorylation in histone-exposed HUVECs. Therefore, we speculate that the protective effect of ADSC-exosomes in the context of histone-induced endothelial damage is partial; importantly, we show here that this effect is related to miR-126. Further research is needed for clarification.

ADSC therapies have recently been applied for severe ARDS and sepsis in humans [7, 8], and some phase II trials are currently underway [46]. Recently, ADSC-based studies were also conducted in the context of (coronavirus disease 2019) COVID-19 pneumonia in basic and clinical settings [50]. Since endothelial cell dysfunction is a major cause of life-threatening events in SARS-CoV-2 infection (COVID-19) [51, 52], endothelial protection by ADSCs could be a novel therapeutic approach for severe ARDS of COVID-19.

## Conclusion

In conclusion, we demonstrated that ADSC-exosomes decreased histone-induced endothelial damage in in vitro and in vivo models via the PI3K/Akt signaling pathway. We speculate that the endothelial protective effect of ADSCs may be, in part, an important mechanism of stem cell therapy. Therefore, we believe that our findings provide novel insights for the development and optimization of cell-based therapy for sepsis and ARDS.

## Supplementary information

**Additional file 1: Figure S1.** (a) Flow cytometric analysis of ADSC immunophenotypes. Cells were stained with immunoglobulin G (IgG) isotype-matched control antibodies or antibodies against CD29, CD34, CD44, CD45, or CD90. (b) Expression of the exosome markers CD63, CD9, CD81, and β-actin confirmed by immunoblotting. (c) Size distribution of exosomes determined by dynamic light scattering. The x-axis on a size distribution plot shows the estimated distribution of particle radii (nm), with the y-axis showing the relative percentages. (d) Relative cell viability of HUVECs exposed to histones (0, 25, 50, 75, 100 μg/mL in each) for 4 h was measured by the Cell Titer-Glo luminescent cell viability assay. Each sample was analyzed in triplicates. ^*^p < 0.05, ^**^p < 0.01 vs. control. (e) Schematic of the co-culture experiments of HUVECs with ADSCs using Falcon Cell Culture Inserts. (f) Expression of CD81 marker from media exposed to GW4869 or DMSO. (g) The relative cell viability of HUVECs after co-culture with or without histone-exposed (100 μg/mL) ADSCs or exosomes for 4 h. In the exosome group, exosomes derived from 1 × 10^4^ ADSCs (same as the ADSCs group) were added to the HUVECs’ medium. *N* = 6 in each group. ^**^p < 0.01 vs. control, ^†^*p* < 0.05 vs. histones. (h, i) Representative immunoblots and densitometry analysis of phosphorylated Akt in HUVECs 4 h after exposure to histones, with or without ADSCs and exosomes. *N* = 4 in each group. ^*^p < 0.05 vs. histones.

## Data Availability

The datasets used and analyzed in this study would be made available through the corresponding author upon reasonable request.

## Abbreviations

MSCs: Mesenchymal stem cells
ADSCs: Adipose-derived mesenchymal stem cells
ARDS: Acute respiratory distress syndrome
HUVECs: Human umbilical vein endothelial cells
NETs: Neutrophil extracellular traps
DAMPs: Damage-associated molecular patterns
MOF: Multiple organ failure
PBS: Phosphate-buffered saline
H&E: Hematoxylin and eosin
miR-126: microRNA-126
RT-PCR: Reverse-transcription polymerase chain reaction
COVID-19: Coronavirus disease 2019
