# Supplementary material for: Exosomes from adipose tissue-derived mesenchymal stem cells ameliorate histone-induced acute lung injury by activating the PI3K/Akt pathway in endothelial cells
Source: Stem Cell Res Ther. 2020 Nov 27;11:508. doi: 10.1186/s13287-020-02015-9 (PMC7691956; doi:10.1186/s13287-020-02015-9)
Supplement: Supplementary file 1 — Additional file 1: Figure S1. (a) Flow cytometric analysis of ADSC immunophenotypes. Cells were stained with immunoglobulin G (IgG) isotype-matched control antibodies or antibodies against CD29, CD34, CD44, CD45, or CD90. (b) Expression of the exosome markers CD63, CD9, CD81, and β-actin confirmed by immunoblotting. (c) Size distribution of exosomes determined by dynamic light scattering. The x-axis on a size distribution plot shows the estimated distribution of particle radii (nm), with the y-axis showing the relative percentages. (d) Relative cell viability of HUVECs exposed to histones (0, 25, 50, 75, 100 μg/mL in each) for 4 h was measured by the Cell Titer-Glo luminescent cell viability assay. Each sample was analyzed in triplicates. *p < 0.05, **p < 0.01 vs. control. (e) Schematic of the co-culture experiments of HUVECs with ADSCs using Falcon Cell Culture Inserts. (f) Expression of CD81 marker from media exposed to GW4869 or DMSO. (g) The relative cell viability of HUVECs after co-culture with or without histone-exposed (100 μg/mL) ADSCs or exosomes for 4 h. In the exosome group, exosomes derived from 1 × 10^4^ ADSCs (same as the ADSCs group) were added to the HUVECs’ medium. N = 6 in each group. **p < 0.01 vs. control, †p < 0.05 vs. histones. (h, i) Representative immunoblots and densitometry analysis of phosphorylated Akt in HUVECs 4 h after exposure to histones, with or without ADSCs and exosomes. N = 4 in each group. *p < 0.05 vs. histones. [file 13287_2020_2015_MOESM1_ESM.docx]

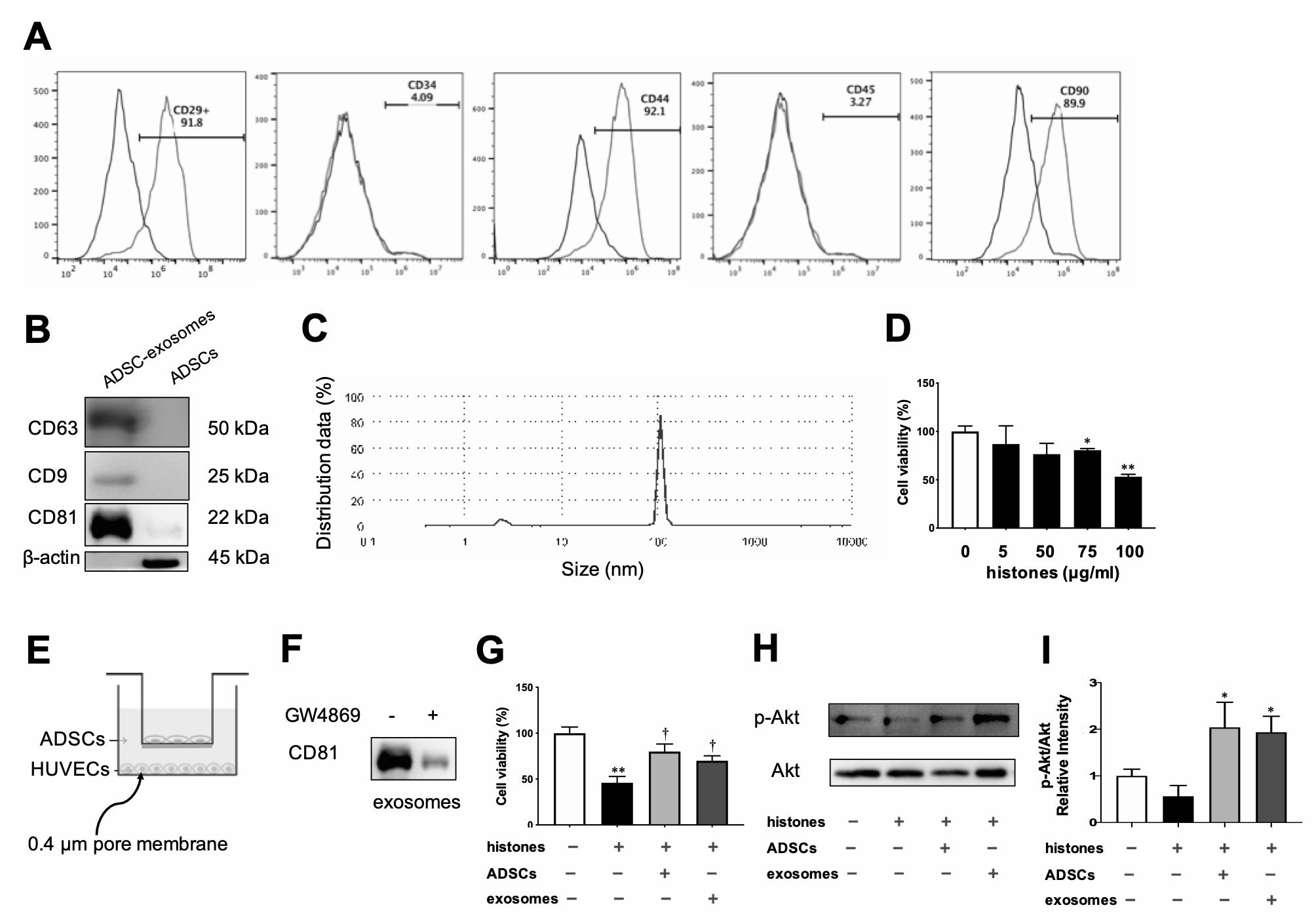


**Supplementary Figure 1: Figure S1.** (**a)** Flow cytometric analysis of ADSC immunophenotypes. Cells were stained with immunoglobulin G (IgG) isotype-matched control antibodies or antibodies against CD29, CD34, CD44, CD45, or CD90. (**b)** Expression of the exosome markers CD63, CD9, CD81, and β-actin confirmed by immunoblotting. (**c)** Size distribution of exosomes determined by dynamic light scattering. The x-axis on a size distribution plot shows the estimated distribution of particle radii (nm), with the y-axis showing the relative percentages. **(d)** Relative cell viability of HUVECs exposed to histones (0, 25, 50, 75, 100 μg/mL in each) for 4 h was measured by the Cell Titer-Glo luminescent cell viability assay. Each sample was analyzed in triplicates. ^*^*p*<0.05, ^**^*p*<0.01 vs. control. **(e)** Schematic of the co-culture experiments of HUVECs with ADSCs using Falcon Cell Culture Inserts. **(f)** Expression of CD81 marker from media exposed to GW4869 or DMSO. **(g)** The relative cell viability of HUVECs after co-culture with or without histone-exposed (100 μg/mL) ADSCs or exosomes for 4 h. In the exosome group, exosomes derived from 1×10^4^ ADSCs (same as the ADSCs group) were added to the HUVECs’ medium. N=6 in each group. ^**^*p*<0.01 vs. control, ^†^*p*<0.05 vs. histones. **(h, i)** Representative immunoblots and densitometry analysis of phosphorylated Akt in HUVECs 4 h after exposure to histones, with or without ADSCs and exosomes. N=4 in each group. ^*^*p*<0.05 vs. histones.
